# Enhanced Nuclear Magnetic Resonance Spectroscopy with Isotropic Mixing as a Pseudodimension

**DOI:** 10.1021/acs.analchem.2c01471

**Published:** 2022-06-13

**Authors:** Dariusz Gołowicz, Alexandra Shchukina, Krzysztof Kazimierczuk

**Affiliations:** † Centre of New Technologies, University of Warsaw, Banacha 2C, 02-097 Warsaw, Poland; ‡ Faculty of Chemistry, University of Warsaw, Pasteura 1, 02-093 Warsaw, Poland

## Abstract

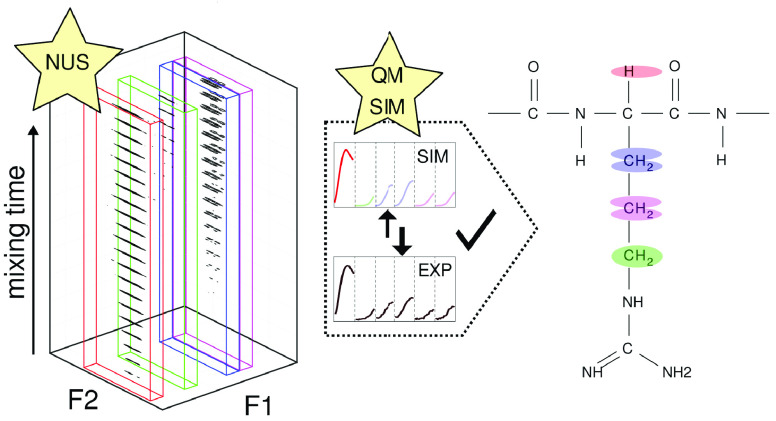

Chemical analysis based on liquid-state nuclear magnetic resonance spectroscopy exploits numerous observables, mainly chemical shifts, relaxation rates, and internuclear coupling constants. Regarding the latter, the efficiencies of internuclear coherence transfers may be encoded in spectral peak intensities. The dependencies of these intensities on the experimental parameter that influences the transfer, for example, mixing time, are an important source of structural information. Yet, they are costly to measure and difficult to analyze. Here, we show that peak intensity build-up curves in two-dimensional total correlation spectroscopy (2D TOCSY) experiments may be quickly measured by employing nonuniform sampling and that their analysis can be effective if supported by quantum mechanical calculations. Thus, such curves can be used to form a new, third pseudodimension of the TOCSY spectrum. Similarly to the other two frequency dimensions, this one also resolves ambiguities and provides characteristic information. We show how the approach supports the analysis of a fragment of protein Tau Repeat-4 domain. Yet, its potential applications are far broader, including the analysis of complex mixtures or other polymers.

## Introduction

Nuclear magnetic resonance (NMR) spectroscopy is widely used in many branches of chemical analysis. It manipulates nuclear spins and measures the response of a sample as a free induction decay (FID) signal. The FID has the form of oscillations decaying in time, so its Fourier transform yields a spectrum with resonance peaks described by Lorentzian functions. The most informative spectral parameters are peak positions and volumes, the former dependent on a molecular structure and the latter proportional to the number of nuclei resonating at a given frequency.

Two-dimensional (2D) NMR, in turn, yields peaks in a spectrum that are described by two coordinates. They can be related to various types of spin interactions in a molecule. The spectral peak heights encode the information about the strength of a coupling. An additional dimension also enhances peak dispersion: When peaks in a 1D spectrum overlay, the second dimension will most probably separate them. 2D NMR is extensively applied in determining molecular structures^1^ and in identifying chemical compounds, for example, components of natural mixtures.^2^

In many research tasks, we acquire a series of spectra varying certain parameters in-between. Usually, we vary an environmental condition^3−6^ or a pulse-sequence parameter.^7−15^ Such a series of spectra can be regarded as one object with a higher dimensionality. A series of 1D spectra thus forms a 2D object; the most well-known example here is diffusion-ordered spectroscopy (DOSY).^9^ A series of 2D spectra constitutes a 3D object, for example, as in nuclear overhauser effect spectroscopy (NOESY) with variable mixing times.^14^ In both cases, the varied parameter forms an extra (second or third) pseudodimension.

Any additional dimension, be it a “normal” Fourier one or a pseudodimension, brings in two advantages for the spectral analysis. First, it enhances the peak dispersion. Second, an additional dimension helps with the peak assignment. When peak parameters from a lower-dimensional spectrum are not enough to state which group of nuclei have yielded a given peak, one more dimension may make up for the missing information. For example, overlapping resonances in 2D ^15^N HSQC spectrum of an unfolded protein are often well separated in the third (CO) dimension of a 3D HNCO spectrum.^7^

In this work, we research the possibilities arising from the incrementation of isotropic mixing time in total-correlation spectroscopy (TOCSY). We show that it can be used to form a spectral dimension providing both the mentioned benefits: higher resolution and a unique peak-characteristic profile.

TOCSY spectra are widely used in chemical analysis as they present (at least theoretically) the correlations between all the coupled nuclei within the entire spin system. In the case of peptides, the amino acid residues form separate spins systems and thus TOCSY crosspeaks usually form amino-acid-specific patterns.^16,17^ The key element of a TOCSY pulse sequence is an isotropic mixing block, implemented with multipulse decoupling sequences (e.g., DIPSI^18^ or MLEV type^19^). During this block, the system (ideally) evolves under a strong coupling Hamiltonian alone, and the transfer proceeds through the whole scalar coupling network.

In practice, however, the coherence transfer under isotropic mixing depends on the *J*-couplings present in the particular spin system as well as on its topology. Such transfer dependencies can be determined by measuring a series of TOCSY experiments with incremented isotropic mixing times, keeping in mind possible distortions caused by a rotating frame nuclear Overhauser effect,^20^ unwanted coherences,^21^ and the signal loss due to *T*_1ρ_ relaxation. TOCSY–transfer curves have already found many interesting applications. For instance, their analysis made it possible to estimate *J*-coupling values in sugar rings^22^ and small biomolecules,^23,24^ identify polyaromatic spin systems in crude gas–oil mixtures,^25^ and assign resonances of small molecules in complex mixtures^26^ and monosacharide units in oligo- and polysaccharides.^27,28^

Knowing the spin-system topology and assuming the *J*-coupling constants, one may calculate the magnetization evolution during the isotropic mixing conditions. For the simple spin systems, the analytical equations have been delivered^29−31^ and used for isotropic transfer optimization.^32^ For the “mechanics” of TOCSY transfer, see also ref (33). However, simulating TOCSY transfer for complex spin systems requires numerical calculations based on a density matrix evolution. Although these calculations are computationally demanding, nowadays they can be carried out efficiently, even for big spin systems using dedicated software, for example, Spinach.^34^

The measurement of intensity build-up curves in a series of 2D TOCSY spectra is lengthy. This is caused by the conventional time-domain sampling strategy based on the Nyquist-Shannon theorem,^35^ which requires the sampling rate to be equal to the spectral bandwidth. For typical bandwidths of ca. 7–10 kHz (^1^H dimension on our 700 MHz spectrometer), thousands of sampling points are needed to reach evolution times, that provide natural, relaxation-determined line widths. High spectral resolution is particularly necessary for spectra of complex molecules, such as the peptide studied in this work. Since every point in the indirect dimension takes several seconds to acquire, the measurement of a single high-resolution 2D spectrum sometimes lasts for tens of hours,^36^ making the serial acquisition of several 2D spectra merely impossible. Fortunately, the approach known as nonuniform sampling (NUS) is a well-established method to accelerate a measurement by omitting major parts of data during the acquisition and reconstructing them afterward using sophisticated mathematical algorithms.^37^

One of the most commonly used NUS methods is compressed sensing (CS). It reconstructs the missing data points by assuming that the resulting spectrum is sparse, that is, that the number of significant spectral points (peaks) is relatively low. This can be done by convex optimization of a simple loss function by taking into account two assumptions about the resulting spectrum: accordance with the experimental data and maximum compressibility.^38,39^ The most commonly applied CS algorithms are iterative soft thresholding^38,40^ and iteratively reweighted least squares.^38,41^

In this article, we apply the NUS/CS approach to accelerate the measurement of transfer curves in 2D TOCSY spectra, effectively creating a pseudo-3D experiment. The shapes of these curves are nuclei-specific and can be used to identify peaks or detect peak overlap. To match curves with the nuclei, the TOCSY transfer has to be effectively simulated. We demonstrate that it can be done using Spinach software^34^ and numerous protein structures deposited in the Protein Data Bank (PDB).^42^ We show the application of the method in assisting the assignment of ^1^H side-chain peaks in an unlabeled, 32-residues long peptide (Tau protein 4-repeat domain).

## Experimental Section

### Sample Preparation

We prepared the sample by dissolving Tau Repeat-4 domain peptide (unlabeled, trifluoroacetate salt form, CPC Scientific) in phosphate-buffered saline (10% D_2_O, pH 6.5) to the concentration of 1 mM.

### Data Acquisition

We acquired a series of 21 zero-quantum filtered ^1^H–^1^H *z*-TOCSY spectra with varied DIPSI-2 mixing sequence^18^ time. The duration of a single supercycle of DIPSI-2 was 3.425 ms. To extend the isotropic mixing time in consecutive spectra, we incremented the number of supercycles in the range from 2 to 22, which corresponded to the mixing time from 6.85 to 75.35 ms. We acquired each 2D TOCSY spectrum with a spectral width of 16.38 ppm in the direct dimension and 10.00 ppm in the indirect dimension, 512 increments in the indirect dimension, 4 scans per increment (8 scans including quadrature detection), and the direct acquisition time of 0.5 s. The total acquisition time of 21 spectra was ca. 40 h. We acquired the data using an Agilent 700 MHz DirectDrive2 NMR spectrometer equipped with a room-temperature HCN probe at 298 K. Raw experimental data and processing scripts are available at DOIs 10.5281/zenodo.6412801 and 10.5281/zenodo.6563108, respectively.

### Data Processing

We subsampled all the acquired 2D TOCSY FIDs down to 75%, 50%, 37.5%, 25%, 18.75%, 12.5%, and 6.25% of the full Nyquist grid in the indirect dimension using exponentially weighted sampling schedules generated with the *nussampler* program from the MddNMR package.^43^ The subsampled signals corresponded to ca. 30 h (75%), 20 h (50%), 15 h (37.5%), 10 h (25%), 7.5 h (18.75%), 5 h (12.5%), and 2.5 h (6.25%) acquisition time. Then we reconstructed the missing points in each subsampled data set using Iterative Soft Thresholding (IST) algorithm (1000 iterations). We applied a Virtual Echo preprocessing^44^ step to increase the signal sparsity before the reconstruction. We performed the preprocessing and data reconstruction with the MddNMR software^43^ on NMRbox.^45^ Finally, we obtained the TOCSY-transfer curves from the original and reconstructed TOCSY spectra by measuring 2D peak volumes with Peakipy software.^46^

### Simulations

In our analysis of the TOCSY-transfer curves, we compared the experimental and simulated data to improve the side-chain assignment in a Tau-R4 peptide. We simulated the TOCSY-transfer curves for side chains in 1188 peptides and small proteins deposited in the Protein Data Bank^42^ (PDB) using the Spinach simulation library for MATLAB (ver. 2.4.5157).^34^ We took PDB entries that had NMR experimental data, had sequence lengths between 5 and 120 residues, were not complexed or assembled into bigger structures, and were deposited between 2010-01-01 and 2021-12-15.

From the PDB coordinates of all models within the entry, we estimated the vicinal ^3^*J*_HH_ couplings for side-chain spin systems using Karplus equations^47,48^ and assumed ^2^*J*_HH_ = −12 *Hz* for the geminal couplings. This was done in Spinach by calling read_pd1b_pro and guess_j_pro functions. Although geminal couplings for methylene groups within amino acid residue spin systems may differ,^49^ setting a single arbitrary value for ^2^*J*_HH_ should not impact simulated TOCSY-transfer curves in our study. For each residue, we simulated the TOCSY transfer by defining the initial state of the spin system as H^N^ in-phase magnetization and tracking its evolution in the Liouville space, assuming scalar coupling interactions only. Because the peptide used in our study is disordered, we narrowed down the simulated data to residues not involved in the formation of secondary structure elements (26,766 residues). After such a filtering, we prepared reference TOCSY-transfer curves for each residue type by averaging out simulated data. Then we compared them to the experimental data. Although in most of cases such a straightforward approach was sufficient, a more sophisticated procedure is also possible and we found it necessary for several residues in our peptide. Namely, we searched for the simulated TOCSY curve for a given residue type that was the closest to the experimental one (in terms of the least residual of their subtraction). In a real spectrum, the peak overlap may lead to unusual TOCSY crosspeak patterns; therefore, we tested various combinations of simulated nucleus-specific transfer curves to recognize such patterns and assign overlapped peaks.

## Results and Discussion

The key benefits of the proposed technique include nuclei identification based on the shape of the build-up curve and peak overlap detection. To make the method feasible, we accelerate it by the application of NUS. These crucial aspects of signal processing and analysis are discussed below.

### Application to Tau-R4 Peptide

**Figure 1:**
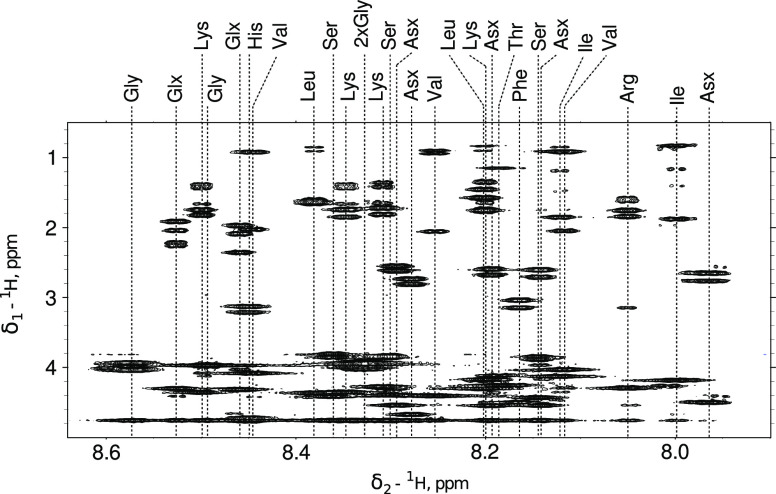
Amide ^1^H TOCSY spectral region for Tau-R4 peptide. The spectrum was obtained by adding 21 TOCSY spectra acquired for different isotropic mixing times (6.85–75.35 ms). Dashed lines indicate H^N^ chemical shifts of the observed residues and their crosspeak patterns.

Resonances of all amide protons in the studied peptide fall within a 0.7 ppm range, as Figure 1 shows. Such a low ^1^H chemical shift dispersion is typical for unstructured peptides and requires extraordinary spectral resolution. On the positive side, the spins in unfolded peptides relax more slowly than those in the structured ones; thus, the TOCSY-transfer curves can be sampled for higher mixing times.^50^ The assignment of ^1^H peaks to particular side-chain protons can be a challenging task, even with a relatively high field spectrometer, such as the one at our disposal. Typically, for unlabeled peptides, the choice of two-dimensional techniques is limited. The combination of homonuclear techniques (TOCSY, COSY, and NOESY) is the only feasible approach^16^ because the heteronuclear methods (e.g., HCCH-TOCSY) are too insensitive. In the current study, we found the extra TOCSY dimension useful to confirm the assignment of side-chain ^1^H NMR signals in several residues of Tau-R4 peptide.

It is often observed that crosspeak patterns for leucine and lysine residues vary significantly: Their crosspeaks may overlay in different combinations or exchange positions. It happens because their β, γ, and δ protons appear at very similar chemical shifts.^51^ In particular, these residues revealed unusual crosspeak patterns in the studied Tau-R4 peptide. We employed TOCSY-transfer curves to detect a potential peak overlap and confirm the peak assignment of these residues.

**Figure 2:**
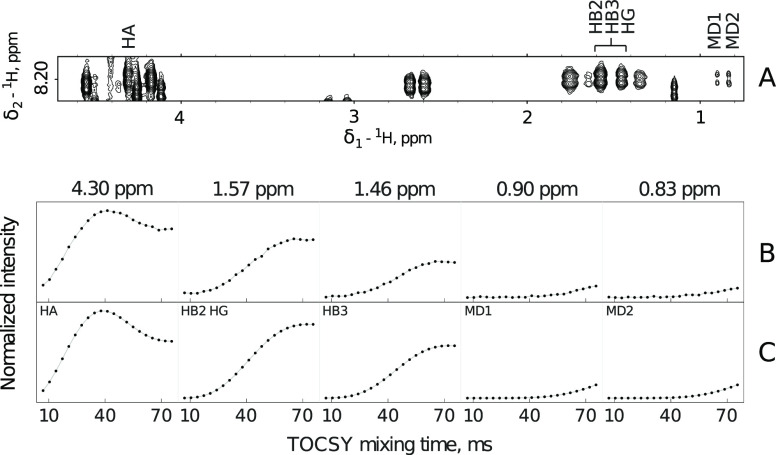
Five-peaks TOCSY pattern (A) for leucine residue in Tau-R4 peptide and the corresponding experimental (B) and simulated (C) TOCSY-transfer curves. The chemical shifts of the analyzed peaks are shown at the top of panel B. Panel C shows the combination of the simulated nucleus-specific TOCSY-transfer curves that matches experimental data. The information on the co-added nucleus-specific curves is in the upper-left corner of each window in panel C. The reference simulated curves were obtained by averaging simulated curves for 1758 leucine residues not involved in secondary structure elements, according to the PDB library.

Using different combinations of nucleus-specific TOCSY-transfer curves for leucine allowed us to reproduce experimental data and confirm that the signal of one of the β protons is overlapped with the signal of γ proton (see Figure 2) and exclude the possibility of H_β2_ and H_β3_ overlap. However, the configurational assignment of leucine’s methylene protons with the analysis of TOCSY transfer is difficult as the build-ups of H_β_ signals show only subtle differences for gauche and trans configurations.^52^

**Figure 3:**
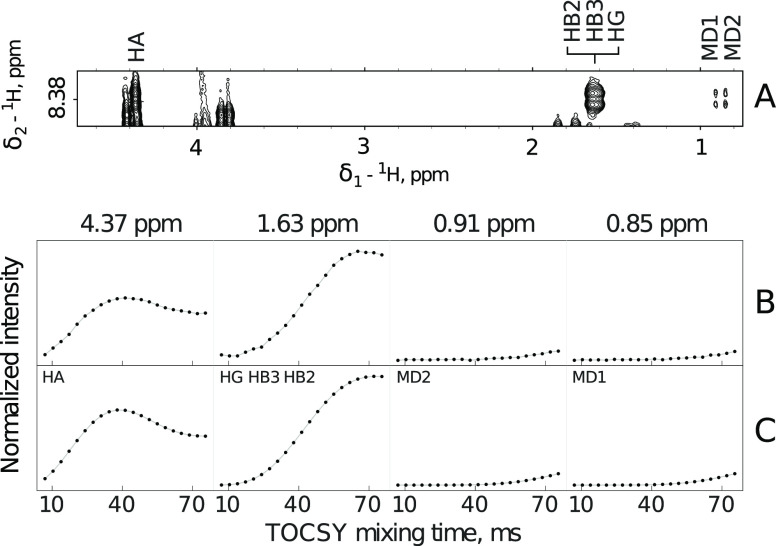
Four-peaks TOCSY pattern (A) for leucine residue in Tau-R4 peptide and the corresponding experimental (B) and simulated (C) TOCSY-transfer curves. The chemical shifts of the analyzed peaks are shown at the top of panel B. The simulation assumed the overlap of H_β_ and H_γ_ protons as indicated in the upper-left corner of the second (from left to right) window in C. The simulated curves were obtained based on 1758 leucine spin systems not involved in secondary structure elements from the PDB library.

Another leucine showed a different crosspeak pattern with one broad peak in a 1–3 ppm region. An isotropic mixing dimension confirms that two H_β_ and one H_γ_ protons contribute to this peak. Summing nucleus-specific curves for H_β_ and H_γ_ protons nicely reproduced TOCSY buildup of the broadened peak at 1.63 ppm, as shown in Figure 3).

**Figure 4:**
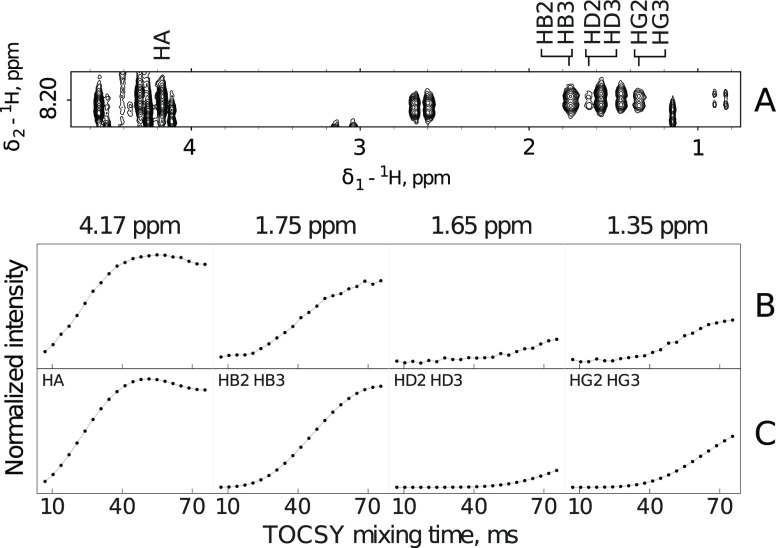
Four-peaks TOCSY pattern (A) for lysine residue and the corresponding experimental (B) and simulated (C) TOCSY-transfer curves. The chemical shifts of the analyzed peaks are shown at the top of panel B. The simulated data assumed the overlap of H_β2_ and H_β3_, H_γ2_ and H_γ3_, and H_δ2_ and H_δ3_ protons, as indicated in panel C. The set of curves matching the experimental data was selected from the library of TOCSY curves containing 2031 lysine spin systems not involved in secondary structure elements.

Another example of residue where side-chain ^1^H NMR peaks appear within a relatively narrow spectral range is lysine. Among four lysine residues present in Tau-R4 peptide, three of them showed a typical crosspeak pattern with unique chemical shifts for each of the H_β_ and H_γ_ protons. The other one showed the number of crosspeaks reduced by two. We assigned the signals in both lysine crosspeak patterns by analyzing the new pseudodimension. The experimental and simulated TOCSY buildups showed that, for lysine with a reduced number of peaks, two H_β_, two H_γ_, and two H_δ_ protons yield three peaks caused by overlap, as indicated in Figure 4.

**Figure 5:**
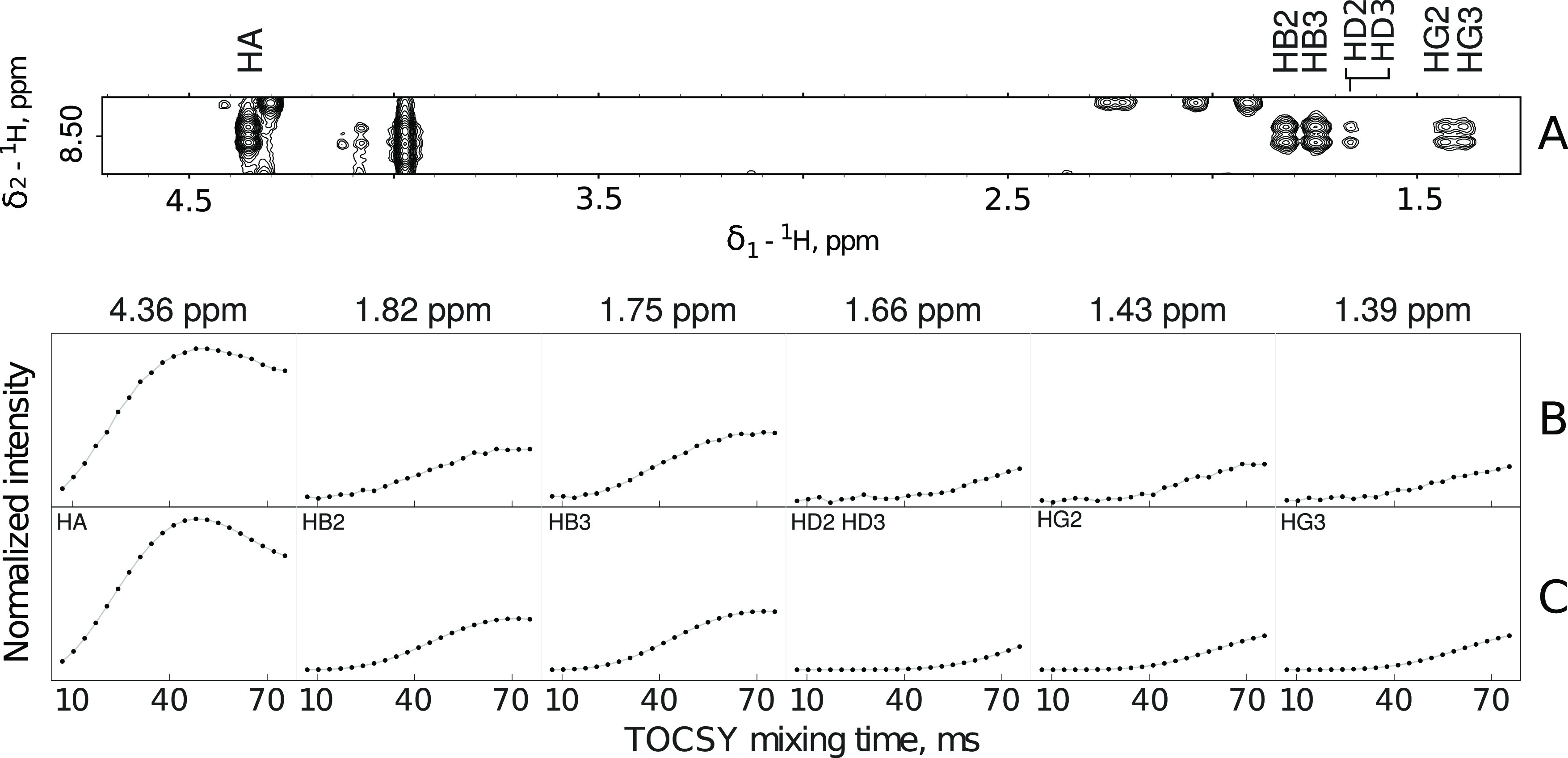
Common six-peaks TOCSY pattern (A) for lysine residue and the corresponding experimental (B) and simulated (C) TOCSY-transfer curves. The chemical shifts of the analyzed peaks are shown at the top of panel B. The simulated data assumed overlap of H_δ2_ and H_δ3_ protons, as indicated in panel C. The set of curves matching experimental data was selected from the library of TOCSY curves containing 2031 lysine spin systems not involved in secondary structure elements.

We performed a similar analysis for one of the lysines with a typical crosspeak pattern to further demonstrate that the simulated TOCSY curves match the experimental data well and can increase assignment reliability (see Figure 5). In this case simulations confirmed that each of the four peaks correspond to one of the H_β_ and H_γ_ protons.

Importantly, all these examples do not take the relaxation or the coherence loss into account for the simulations and still provide a good agreement with the experimental data. Furthermore, one may potentially combine the acquisition of the TOCSY-transfer dimension with multiplet collapsing methods^53,54^ for easier identification of overlapping peaks.

### Accelerated Measurement of TOCSY-Transfer Curves

As demonstrated, matching the experimental and simulated TOCSY-transfer curves significantly supports the spectral analysis and the resonance assignment. However, collecting a long series of 2D spectra to obtain precisely described TOCSY-transfer profiles takes tens of hours. For example, a pseudo-3D TOCSY experiment for Tau-R4 peptide costed us about 40 h of experimental time. Thus, to accelerate the acquisition, we propose to employ NUS, followed by the signal reconstruction using the CS method.

**Figure 6:**
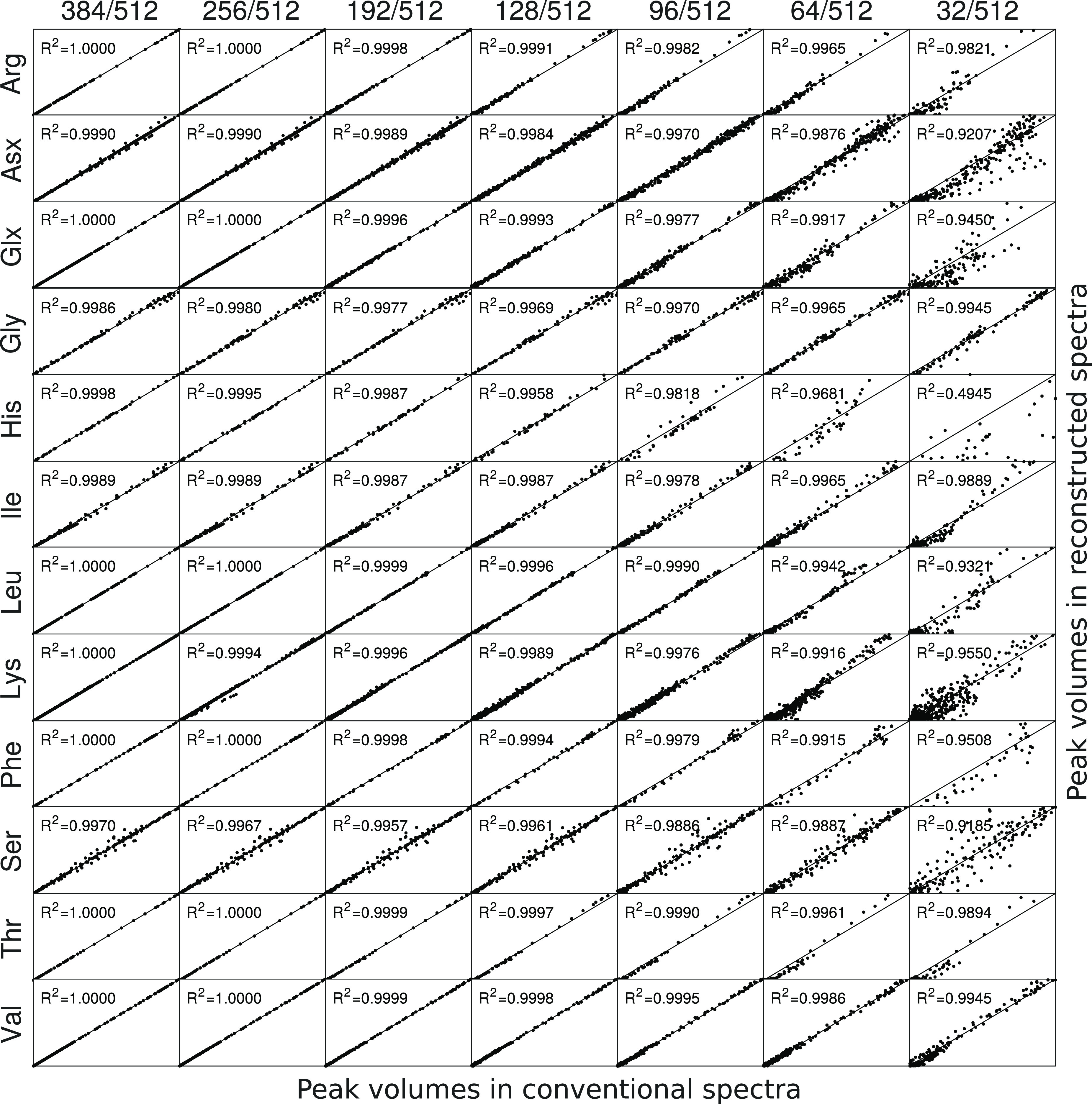
Correlations between normalized cross-peak volumes in conventional spectra (512/512 sampling) and reconstructed spectra (384/512, 256/512, 192/512, 128/512, 96/512, 64/512, and 32/512 sampling levels) plotted for each residue type (row-wise). Subplots contain cross-peak volumes for each residue type in the 21 TOCSY spectra acquired for different isotropic mixing times. The horizontal axis in each subplot corresponds to the cross-peak volumes measured in conventional, fully sampled spectra, whereas the vertical axis shows peak volumes in reconstructed NUS spectra.

The nonlinearity of the CS reconstruction, particularly pronounced for low sampling levels, is a known problem.^55^ This unwanted effect has to be minimized because the unbiased shapes of build-up curves are necessary for the feasibility of the method. Thus, we evaluated the reconstruction of TOCSY buildups at different subsampling levels by comparing peak volumes in the reconstructed spectra to those from the uniformly sampled spectra. The results of this test are shown in Figure 6.

We observed that TOCSY-transfer curves can be reliably reproduced at sampling levels as low as 18.75% of the Nyquist grid (see the “96/512” column in Figure 6). It means that the acquisition can be accelerated about fivefold. In our study, it corresponds to shortening the experimental time from ≈40 to ≈7.5 h. This significant reduction of acquisition time allows for collecting data overnight and makes the experiment practical to use, even for difficult samples such as our peptide. Because the total time required to collect the series of spectra for the peptide resonance assignment (^15^N HSQC, ^13^C HSQC, COSY, TOCSY, and NOESY) reaches several days, an extra few hours seem to be a reasonable price for the amount of information gained.

**Figure 7:**
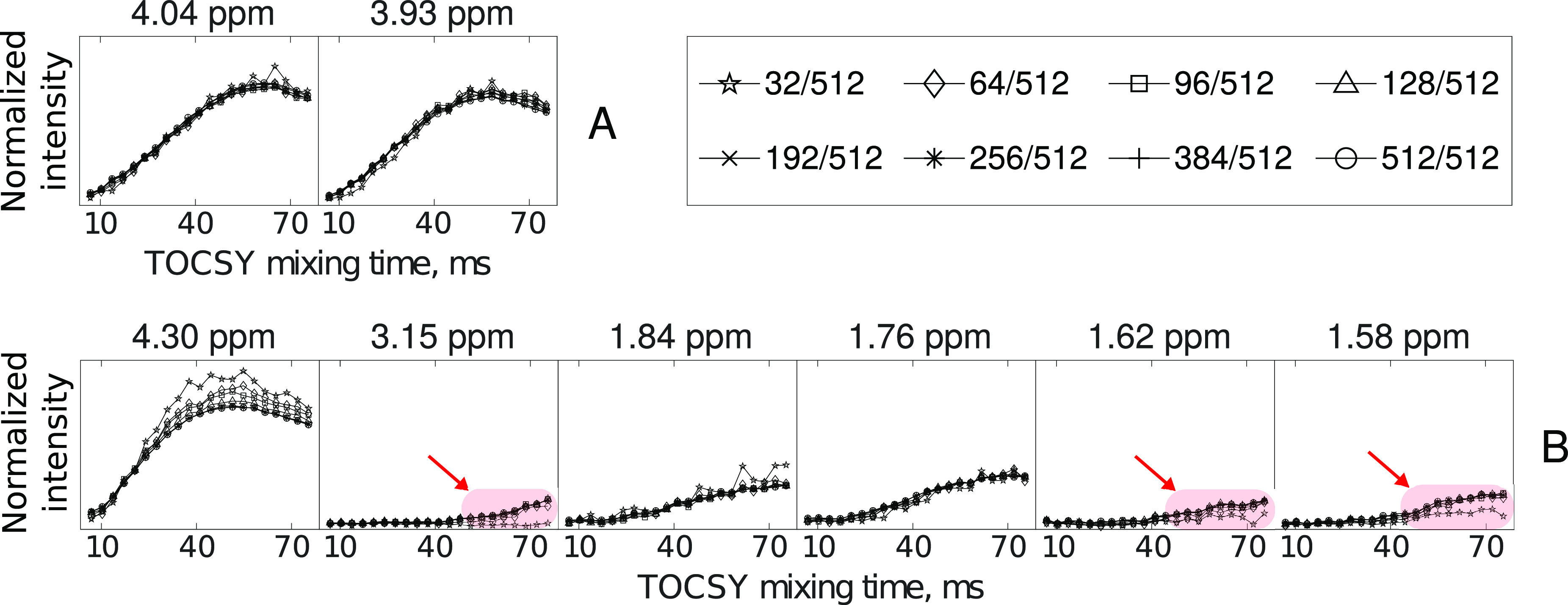
TOCSY-transfer curves for glycine at H^N^ chemical shift of 8.57 ppm (A) and arginine at H^N^ chemical shift of 8.05 ppm (B) obtained from spectra reconstructed at different subsampling levels (32/512, 64/512, 96/512, 128/512, 192/512, 256/512, and 384/512) and from conventional (512/512) spectra. The chemical shifts of the analyzed peaks are shown at the top of each subplot. The red arrows indicate the regions of the curves where the reconstruction was poor at low sampling levels.

Figure 6 also indicates that TOCSY buildups for smaller spin systems (e.g., glycine) were reproduced well, even at the lowest tested sampling level, that is, 32/512, whereas the reconstruction for bigger spin systems, such as Arg, Ile, Leu, or Lys, failed. This is in agreement with the CS theory, which states that the minimum number of points required for successful reconstruction is proportional to *K* log(*N*). Here, *K* is the number of significant spectral points and *N* is the length of the vector (here, *N* = 512 *t*_1_ points; see ref (55) for detailed examples). At low sampling levels, the reconstruction often fails for weak signals of bigger spin systems. This usually refers to (H_γ_ and H_δ_) protons as the coherence transfer from H^N^ to these distant protons is generally weak for isotropic mixing times under 100 ms.^52^ An example of correct and bad TOCSY-curves reconstruction at a low sampling level for small and big spin systems is shown in Figure 7.

The reconstruction of TOCSY curves for glycine (see Figure 7A) at a 32/512 sampling level matches almost perfectly with the results from the conventional 512/512 sampling. On the other hand, at the same sampling level, CS failed to reconstruct weak H_γ_ (1.62 and 1.52 ppm) and H_δ_ (3.15 ppm) signals of arginine (see red arrows in Figure 7B). As shown previously,^55^ at low sampling levels, CS reconstructs only the most intense H_α_ and H_β_ signals. As the CS reconstruction is performed trace by trace in the indirect dimension, the performance of CS is generally worse when the number of peaks grows, for example, because of similar H^N^ chemical shifts of two different residues.

**Figure 8:**
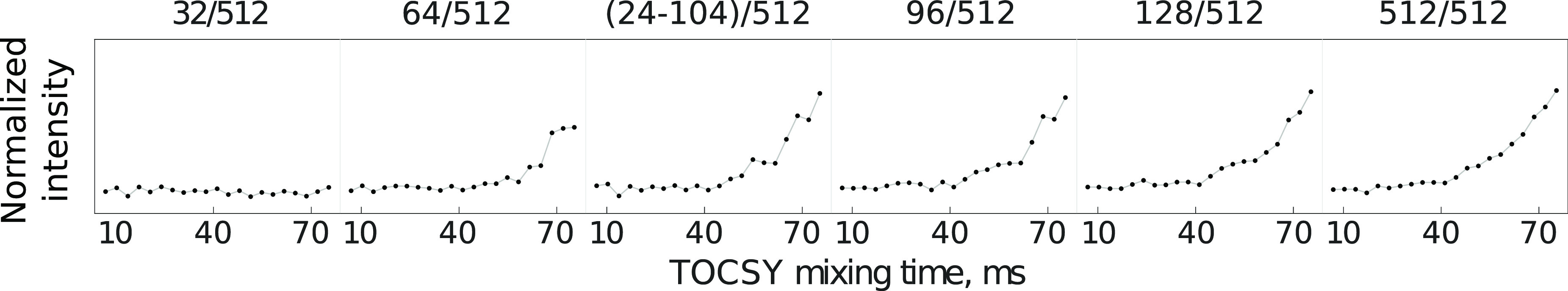
Experimental TOCSY build-up curves for a H_δ_ crosspeak of arginine (8.05, 3.15 ppm) obtained at different NUS levels. The applied subsampling levels are shown at the top of the corresponding subplots. The subplot labeled 512/512 corresponds to a series of conventional 2D TOCSY spectra, whereas the subplot labeled (24–104)/512 refers to the sparsity-matched approach. In the sparsity-matched sampling, the first point of the build-up curve corresponds to 24/512 NUS samples and the last point to 104/512 NUS samples.

An interesting feature of pseudo-3D TOCSY acquisition in terms of CS reconstruction is that the spectral sparsity decreases with the length of the isotropic mixing block. We realized that, for a given total experimental time, it is beneficial to decrease the number of NUS points for lower mixing times and collect more samples for longer mixing times. We demonstrate the benefits of this approach on a weak arginine crosspeak at 3.15 ppm (H_δ_) from Figure 7B. Notably, in such a “sparsity-matched” approach, we incremented the number of NUS samples per mixing time from 24/512 to 104/512 with the step of 4. As can be seen in Figure 8, the shape of the curve for the arginine crosspeak is reproduced better from the sparsity-matched sampled data (24–104)/512 than from the data with constant 64/512 sampling, although both data sets contained the same total amount of NUS samples (1344).

Additional to resolution and spectral information gains, the presented method also provides sensitivity benefits, similar to the previously published swept-coherence transfer (SCoT) method.^56^ Namely, a dense sampling of mixing times in pseudo-3D TOCSY prevents the potential missing of crosspeaks, which often occurs in single-2D TOCSY spectra acquired with too short or too long mixing times. On the other hand, because many spectra are needed to describe the shape of the intensity build-up curve, the sensitivity of each spectrum in a series is accordingly smaller than that in a single spectrum acquired over the same time.

The NOESY variant of an approach presented here deserves a comment. Such a method has been proposed in the past by our group.^15^ Contrary to the TOCSY case, the NOESY mixing time can be set to a different value for each *t*_1_ point, allowing for the use of time-resolved NUS.^57^ In this way, one obtains a “continuous” intensity build-up curve with a well-described linear part that can be used to calculate internuclear distances. Yet, significant differences between the magnitudes of the diagonal and off-diagonal peaks make NOESY very challenging for NUS.^58^ Moreover, the shape of the intensity build-up curves in NOESY is not as “sophisticated” as that in TOCSY and thus does not require a dense sampling. Actually, two well-placed points (mixing time values) provide sufficient information, making it possible to determine internuclear distances.^14^

Finally, we would like to make a remark on the possibility of using methods other than CS. Some NUS algorithms allow the co-processing of 2D spectra in a series by gathering them into one pseudo-3D object and applying 3D processing. Such methods employ extra assumptions about the model of changes in the third pseudodimension. For example, the recently introduced methods based on the Radon transform and its variants assume simple models of changes of peak positions.^4,59^ If peaks do not move between spectra, the method of multidimensional decomposition (MDD) is applicable. Apparently, this would be the case of a series of 2D TOCSY spectra. Unfortunately, however, the procedure that greatly enhances the efficiency of MDD for a single 3D spectrum, that is, gathering all off-diagonal peaks into one component,^60^ is not possible for such cases of serial 2Ds when different off-diagonal peaks change their intensities independently. In our hands, the MDD processing of a series of 2D TOCSYs did not give optimal results. Thus, we decided to process our 2D spectra separately using the compressed sensing (CS) method.^38,39^

## Conclusions

Our results show that the TOCSY-transfer curves can be used to form a spectral pseudodimension, providing benefits similar to those of extra Fourier dimensions, that is, the resolution boost and the complement of nucleus-specific information. However, to take full advantage of the concept, we need to sample the mixing time densely and be able to simulate the shape of the build-up curve with high fidelity. The former can be obtained with NUS and effective signal reconstruction methods and the latter requires simulations of strongly coupled spin systems. Both are possible with modern software—MddNMR and Spinach—and open a way to a new type of multidimensional NMR spectroscopy. Although the calculations may take several days, they have to be performed only once, and the obtained library is general for all unstructured peptides. The usability of the isotropic mixing pseudodimensions and the analysis supported by quantum-mechanical simulations reaches far beyond the peptide research discussed in this work. We envisage applications to other complex macromolecules and mixtures where the resolution is an issue. It is worth noting that the build-up curves can be used as a “fingerprint”. For example, when we expect a limited set of compounds in the sample (e.g., in metabolomics), the library of experimentally measured curves can be created and used for compound identification.^26^ Moreover, an extra pseudodimension can be potentially used to enhance the resolution in NMR spectrometers operating at low magnetic fields, for example, benchtop NMR spectrometers.
